# Autophagy and nutrigenomics: a winning team against chronic disease and tumors

**DOI:** 10.3389/fnut.2024.1409142

**Published:** 2024-12-05

**Authors:** Roberto Chiarelli, Fabio Caradonna, Flores Naselli

**Affiliations:** ^1^Department of Biological, Chemical and Pharmaceutical Sciences and Technologies (STEBICEF), University of Palermo, Palermo, Italy; ^2^National Biodiversity Future Center (NBFC), Palermo, Italy

**Keywords:** nutrigenomics, autophagy, dietary-genetic interactions, personalized dietary approaches, nutrigenomic disease strategies

## Abstract

Autophagy, a vital cell process, has garnered attention for its role in various diseases and potential therapeutic interventions. Dysregulation of autophagy contributes to conditions such as metabolic diseases, neurodegenerative disorders, and cancer. In diseases such as diabetes, autophagy plays a crucial role in islet β-cell maintenance and glucose homeostasis, offering potential targets for therapeutic intervention. Nutrigenomics, which explores how dietary components interact with the genome, has emerged as a promising avenue for disease management. It sheds light on how diet influences gene expression and cellular processes, offering personalized approaches to disease prevention and management. Studies have showed the impact of specific dietary components, such as polyphenols and omega-3 fatty acids, on autophagy processes, suggesting their potential therapeutic benefits in neurodegenerative conditions and metabolic disorders. In cancer, autophagy’s dual role in either suppressing tumorigenesis or promoting cancer cell survival underscores the importance of understanding its modulation through dietary interventions. Combined with conventional chemotherapy drugs, dietary compounds show synergistic effects in cancer treatment. Furthermore, phytochemicals such as indicaxanthin have been found to epigenetically regulate genes involved in autophagy, offering novel insights into personalized cancer therapies. This comprehensive review has the aim to study the autophagy in a combined view with nutrigenomics effects of some dietary molecules in maintaining cellular homeostasis and responding to pathological stimuli. Overall, the intersection of autophagy and nutrigenomics effect of bioactive compounds holds promise for developing targeted interventions for various diseases, emphasizing the significance of dietary interventions in disease prevention and management.

## 1 Introduction

Autophagy, a conserved cell process, plays a pivotal role in maintaining cellular homeostasis by recycling damaged organelles and proteins. This self-degradative mechanism is crucial for cellular renewal, adaptation to stress, and overall organismal health. On the other hand, autophagy can mediate a cell death mechanism, independently from apoptosis, and it is often classified as type 2 programmed cell death. Autophagy, derived from the Greek words “*auto*” (self) and “*phagy*” (eating), refers to a cellular recycling process vital for maintaining cellular health and homeostasis. Molecularly, autophagy is regulated by a set of conserved genes called autophagy-related genes (ATGs), which orchestrate the various stages of autophagosome formation and maturation (1). Several key players and signaling pathways regulate autophagy. The mTORx (mammalian target of rapamycin) × pathway is a central regulator that inhibits autophagy in nutrient-rich conditions and promotes it in response to nutrient deprivation or stress (2). Beclin-1, part of the class III phosphatidylinositol 3-kinase complex, plays a crucial role in autophagosome formation. Other proteins such as LC3 (microtubule-associated protein 1A/1B-light chain 3) and p62/SQSTM1 (sequestosome 1) are involved in cargo recognition and degradation within autolysosomes (3).

On the other hand, nutrigenomics investigates the intricate relationship between nutrition, gene expression, and health outcomes. It examines how dietary components and patterns influence gene expression, impacting various physiological processes, including autophagy, metabolism, and ultimately, an individual’s health and susceptibility to diseases (4).

By studying interactions between nutrients and gene regulation, nutrigenomics aims to uncover personalized dietary recommendations tailored to an individual’s genetic makeup, leading to better health outcomes and disease prevention (5).

Emerging research indicates a bidirectional relationship between diet/nutrients and autophagy processes (6). Certain dietary components, such as polyphenols and omega-3 fatty acids, have been shown to modulate autophagy, influencing cellular health and disease susceptibility (6). Conversely, autophagy plays a role in nutrient sensing and metabolism, highlighting its significance in mediating the effects of dietary interventions on cellular function and overall health.

The interplay between autophagy and diet bioactive compound gene regulations significantly influences various disease contexts. Autophagy dysregulation has been implicated in various diseases, showcasing its pivotal role in maintaining cellular homeostasis. Neurodegenerative diseases such as Alzheimer’s, Parkinson’s, and Huntington’s disease exhibit impaired autophagy clearance, leading to the accumulation of toxic protein aggregates (7). In cancer, both excessive autophagy and its inhibition contribute to tumor progression and treatment resistance (8). Metabolic disorders such as type 2 diabetes and obesity also display altered autophagy processes, affecting insulin sensitivity and lipid metabolism (9).

Similarly, emerging evidence underscores how specific dietary components and patterns can modulate autophagy processes, thereby impacting disease susceptibility and progression. Several studies have highlighted the influence of dietary components on autophagy and subsequent disease outcomes. For instance, caloric restriction and fasting have been shown to induce autophagy, promoting longevity and mitigating age-related diseases in model organisms (6). Specific nutrients such as resveratrol, found in red grapes, activate autophagy and may have implications in treating cardiovascular diseases and cancer (10). Conversely, diets high in saturated fats or sugars may inhibit autophagy, contributing to the development of metabolic disorders and cardiovascular diseases (11).

Nutrigenomics interventions have exhibited promise in modulating autophagy-related disease pathways. For instance, personalized dietary recommendations based on genetic profiles have shown efficacy in improving autophagy flux and ameliorating symptoms in individuals with neurodegenerative diseases. Additionally, dietary compounds identified through nutrigenomics studies, such as resveratrol, curcumin and sulforaphane, demonstrate potential in enhancing autophagy and combating inflammation in various diseases, including cancer and inflammatory conditions (7, 12, 13). This review aims to synthesize current understanding and recent advancements in the reciprocal relationship between autophagy and diet bioactive compound gene regulations (nutrigenomics). It intends to elucidate how dietary components influence autophagy processes at a molecular level and the implications of these interactions in various disease contexts. Additionally, this review aims to highlight potential therapeutic avenues and future research directions in the field of nutrigenomics.

## 2 Autophagy: mechanisms and role in pathologies

In recent decades, the role of autophagy in pathogenesis has attracted considerable interest as it could provide useful explanations in the evolution of several diseases (14) and its modulation could help to discover new drugs usable in therapy (15).

Under physiological conditions, autophagy represents an intracellular protective mechanism of self-degradation and the recycling process in eukaryotes (16). The autophagy machinery can remove several damaged organelles (dysfunctional mitochondria, altered endoplasmic reticulum, etc.), intracellular pathogens, and aggregates of proteins destined for degradation (17). Overall, autophagy regulates metabolism, control cell damage, and cell survival (18), modulates cyto-protection and cell death (19).

### 2.1 Defining autophagy and its cellular maintenance functions

Numerous studies have highlighted the role of autophagy in maintaining cellular functions. This is certainly related to the central role of autophagy as the major intracellular degradation system by which cytoplasmic materials are delivered to and degraded in the lysosome (20). The fundamental role of autophagy for maintaining cellular homeostasis is not correlated only to the simple elimination of damaged cellular materials, but instead, to the dynamic recycling system that produces molecules for cellular renovation and energy supply (16).

In general, the definition of the term autophagy refers to the main subtype of the process, macroautophagy, which represents the most widespread form. There are actually three most studied forms of autophagy: macroautophagy, microautophagy and chaperone-mediated autophagy (20).

Macroautophagy is a process involving the lysosomal degradation of large cytoplasmic components (organelles and aggregated proteins), within an autophagy vacuole (autophagosome) delimited by the double phospholipid sheet (of non-lysosomal origin) which subsequently fuses with the lysosome, thus forming the autophagolysosome (16). Once degraded, the simple molecular substances contained in the vacuole are reabsorbed or used by the cells or made available to the organism (21).

Microautophagy is involved in the uptake and degradation of complete regions of the cytoplasm, including proteins and cytoplasmic organelles, directly by lysosomes, without requiring the formation of intermediate autophagy vacuoles. The lysosome, by invagination or protrusion, envelops the cytoplasm and, subsequently, closes to form an internal vesicle which contains the material to be degraded (22).

Microautophagy has traditionally been considered as a form of autophagy aimed at ensuring the protein turnover (23), but other studies (24) also consider it responsible, in part, for peroxisomal degradation.

Chaperone-mediated autophagy can be triggered by numerous cell types for the degradation of cytosolic proteins (25–27). This molecular mechanism is selective because it involves particular groups of proteins and promotes the direct translocation across the lysosomal membrane, without requiring the formation of vacuoles or rearrangement of the membranes. All substrates contain a sequence linked to the pentapeptide KFERQ (26). This motif is recognized by chaperonins, including the HSC70 protein (28) that binds the target proteins. The chaperonin-protein complex binds the lysosomal membrane by interacting with the LAMP-2a (lysosome-associated membrane glycol protein) receptor and lysosomal HSC70 (lys-HSC70) drives the transport within the organelle. The selective feature of this form of autophagy suggests its activation when specific proteins need to be degraded (26, 29).

### 2.2 Autophagy’s role in diseases such as cancer, diabetes, and neurodegenerative disorders

Numerous studies demonstrate the important protective role of autophagy against disease. However, the role of autophagy in some pathologies needs to be characterized because it could act in an ambiguous way, and it is necessary to design treatments based on inhibition or promotion of autophagy.

Early findings indicated a dual role of autophagy in cancer. Several research highlight the different role of autophagy in cancer initiation and progression. In general, autophagy suppresses tumor initiation, but in established tumors it supports uncontrolled cell growth and increased metabolic activities. In addition to dependence related to the tumor stage, autophagy is influenced by specific oncogenic mutations and cellular context (30).

For example, in studies on breast cancer cell lines and primary mammary tumor material, a tumor-suppressive role of autophagy was reported. In particular, frequent allelic loss of *BECN1* (Beclin, a gene that encodes a protein that regulates autophagy) can be related to *BRCA1* (Breast Cancer gene 1) tumor suppressor (31).

As already mentioned, there are different forms of autophagy, and these appear to be implicated in various ways in tumor suppression. For example, mitochondria-selective autophagy (mitophagy) allows the degradation of these damaged organelles. It has been reported that the accumulation of these mitochondria in cells in which key autophagy genes are deleted is related to tumor suppression (32).

On the other hand, there are many scientific data that highlight the role of autophagy in tumor progression. For example, autophagy supports the growth and metabolism of advanced tumors downstream of the activation of various oncogenes and/or inactivation of tumor suppressors (33, 34). Furthermore, autophagy enhances resistance to stress and cytoprotection by eliminating obsolete proteins and organelles, which significantly influences tumor progression (33).

Overall, there is a double face of autophagy in tumorigenesis; it is a tumor suppressor via degradation of potentially oncogenic molecules. Later on, in advanced stages, autophagy promotes the survival of tumor cells.

The interest in studying the relationship between the modulation of autophagy and neurodegenerative diseases is growing because, in many neurodegenerative diseases, an aggregation of misfolded proteins was observed. This represents one of the multiple causes of neuronal cell death (35). Cellular aggregations of misfolded proteins are the most common pathological hallmark of many neurodegenerative diseases, including Alzheimer’s disease, Parkinson’s disease, Huntington’s disease and amyotrophic lateral sclerosis (36).

The study of molecular pathways correlated with autophagy represents a topic of considerable interest also for identifying therapeutic targets for the treatment of these diseases.

Although there are promising experimental results induced by autophagy inductors, treatment (dose and duration) needs to be carefully studied since an increased autophagy activation could cause deleterious effects, promoting an acceleration in the progression of neurodegenerative diseases (35).

Autophagy implication in dysmetabolic pathologies represents an interesting topic, for example in diabetes. Autophagy plays an essential role in the structural maintenance of islet β cells (37). On the other hand, several studies have shown that the regulation of autophagy processes leads to beneficial effects in the treatment of type 2 diabetes (38). Given the considerable interest about the relationship between the autophagy processes and diabetes, the molecular mechanisms underlying autophagy activation in this dysmetabolic pathology will be explored.

### 2.3 Molecular mechanisms underlying autophagy: focus on diabetes

Diabetic mellitus is the most common chronic disease worldwide (39). The most common form of diabetes in adults is type 2, characterized by insulin resistance and islet β cells dysfunction (40). Recent studies have highlighted that loss of function in autophagy can be related to diabetic conditions (41). Since autophagy can have a dual role, both as a survival strategy and as a cell death mechanism, several studies have tried to relate this dual role to the onset of type 2 diabetes.

At basal level, autophagy is important for maintaining of islet β cells. Genetic ablation of atg7, an E1-like ligase that plays a central role in autophagosome biogenesis, promotes islets degeneration and impaired glucose tolerance with reduced insulin secretion (42). *In vivo* studies demonstrated that the levels of the main autophagy protein marker, LC3, were increased when INS-1 cells were exposed to high levels of insulin, demonstrating that the high level of insulin increased autophagy (43). Overall, these studies demonstrate that regulation of autophagy could represent a therapeutic strategy in the treatment of diabetic patients. Abe et al. (44) reported that patients with type 2 diabetes showed high levels of p62/SQSTM1 in islet β cells. P62/SQSTM1 is a protein which resides at the autophagosome membranes. It is an autophagosome cargo protein that targets other proteins that bind to it for selective autophagy, especially under stressful conditions (17).

The accumulation of p62/SQSTM1, which is an adaptor protein interacting with LC3-II, in β cell of diabetic patients may be attributed to either the enhanced or inhibited autophagy flux. Indeed, under normal conditions, p62/SQSTM1 marks proteins destined to autophagy degradation. It increases in conditions of extremely active autophagy, to promote the disposal of the proteins to be degraded (17), alternatively it increases when the autophagy machinery is blocked because, as a consequence, the p62/SQSTM1 protein itself is not degraded and accumulates in cells (16).

In recent years, the interest in the study of molecules modulating autophagy in the treatment of type 2 diabetes has been growing.

Suppression of autophagy inhibitors, such as mTORC1 or PKA (protein Kinase A) (45), induces a fasting-mimicking diet in mice and can promote β cell regeneration (46). Intermittent fasting increases autophagy and inhibits the Notch 1 pathway, increases the expression levels of neurogenin-3 and increases the β-cell mass (47).

Several studies showed the opportunity to use vanadium derivatives as potential therapeutic factors in some diseases, such as obesity, cancer, neurodegenerative and heart disorders. This metal may also be able to reduce the plasma glucose levels of type 1 and 2 diabetes mellitus lacking insulin sensitivity (48, 49). The mechanism is not yet completely clear but using the sea urchin embryo as an experimental model, it has been demonstrated that this metallodrug promotes various modulations relating to cytoprotection (variation in HSPs levels), induction of apoptosis and above all modulation of autophagy phenomena (19, 50).

Results indicated that a direct relationship between type 2 diabetes and autophagy modulation exists and open new research fields for further investigation aimed to discover effective drugs to treat this pathology (38).

## 3 Nutrigenomics: basics and implications in autophagy

### 3.1 Explaining nutrigenomics and its impact on gene expression

Until a few decades ago, it was believed that the genome had no influences from the environment and had no exchanges with the latter. Quantitative genetics, however, had already predicted in the last century, albeit in a non-specific manner, that some characteristics were influenced by the environment, so much so that today we speak of quantitative trait loci precisely to indicate these characteristics. Today, however, it is quite well-known that in the life of all higher eukaryotes, from conception to death, the epigenomes of all tissues and cell types integrate signals from the environment (51). Certainly, if there is an “environment” that we all come into mandatory contact with on a daily basis, it is that of nutrients, of diet. Often, nutrient bioactive molecules change transiently the gene expression, else some effects are persistent and can even be inherited by the next generations. The argument that diet can modulate the epigenome had minted a new genetic/genomic subdiscipline called nutrigenomics or better nutritional epigenetics, which also help to interpret some disease risks, including their prevention (52). Nutrigenomics often use bioinformatics, genomics, molecular biology, molecular medicine, and epidemiology (53).

Once born, many purposes were associated to nutrigenomics: i) defining genomic diversity among various ethnic groups, nutrients bioavailability as well as metabolism (53); ii) creating personalized diets based on the unique metabolic profile of an individual, gut microbiome, and genetic makeup, revolutionizing the field of human nutrition (54); iii) explaining some of the susceptibility for complex diseases, such as the metabolic syndrome, cancer and immune disorders (52).

More recently, it has been also determined that several of these diet-(epi)genome interactions and the involved signal transduction cascades are redox-regulated (51). More specifically, Gabbianelli et al. (55) even reported that some plant-derived substances, acting as epigenetic modulators for broad-spectrum anti-viral activity, will be useful compounds with nutrigenomics effect against future undesirable but probable epidemics due to climate change.

Finally, side to nutrigenomics it is important to consider all the gene-diet interactions studied by nutrigenetics. In fact, the impact of genetic variation on nutrient requirements is equally important in determination of regulation of autophagy processes. Apparently contradictory data on the role of diet in inflammatory bowel disease would be entirely explainable by taking into consideration genetic variability for determined genes responsible, for example, of intolerances (4).

### 3.2 Interconnections between diet bioactive compound and autophagy

**FIGURE 1:**
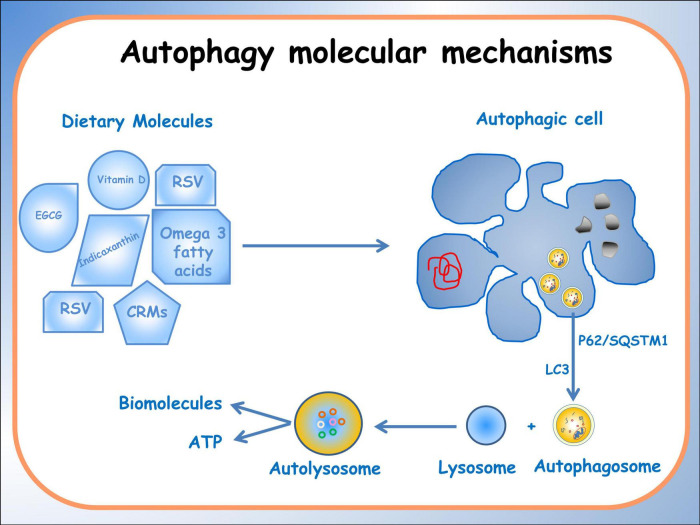
Schematic representation of the effects of different dietary molecules on cellular processes, illustrating their varying actions depending on specific pathologies. The diagram depicts the activation of the autophagic process, highlighting the formation of an autophagosome that encloses organelles and molecules targeted for degradation.

Autophagy is an evolutionarily conserved process critical in maintaining cellular homeostasis. Recently, it was suggested that autophagy, nutrition-induced, exerts a high potential in the prevention and therapies of chronic diseases (56); several examples can be given as demonstration. Sulforaphane and vitamin D, in combination, have a potential application in prostate cancer therapy thanks to their modulating power of the JNK/MAPK (C-jun N-terminal Kinase/Mitogen Activated Protein Kinase) signaling pathway in PC-3 cells (57). Zhou et al. (58) reported some anti-cancer activities of dietary saponins exerted, among other pathways, also via autophagy by regulation of several critical signaling pathways, including MAPK, PI3K/Akt/mTOR, NF-κB, and VEGF/VEGFR. Because several of diet-(epi)genome interactions are redox-regulated (51), the binomial “Diet bioactive compounds -Redox-balance” is important to consider. In fact, it was also reported that the accumulation of reactive oxygen species is the mediator of autophagy in colon cancer cells (59). An extensive methylomic study led by the authors of the present study demonstrated, for the first time, that Indicaxanthin, a phytochemical prick pear-contained has a pro-autophagy potential in human colorectal cancer cells associated with epigenetic changes. In particular, it has been observed that Indicaxanthin can modulate DNA methylation and regulate gene expression (60). Additionally, it exerts significant differential methylation in 39 out of 47 autophagy-related genes, particularly those involved in the late stages and in the fusion stage of autophagy such as *BECN1, MTOR*, *ULK2 (Unc-51Like Autophagy activating Kinase 2), BCL2.2 (B-cell lymphoma)*. Furthermore, *in silico* molecular modeling studies suggested a direct interaction of Indicaxanthin with Bcl-2, which, in turn, influenced the function of Beclin1, a key autophagy regulator (61). Finally, a greater interplay between nutrigenomics effects of dietary compound and autophagy was seen in (Alcohol-Associated Bioactive Dietary Compounds) AABDCs. Caradonna et al. (62) showed a synoptic table reporting biological effects of AABDC components, demonstrating that autophagy is more promoted by the dietary low-dose alcohol. A schematic representation of the effects induced by different dietary molecules on autophagic processes, on specific pathologies has been reported in Figure 1.

### 3.3 How nutrients regulate autophagy through nutritional genomics mechanisms

Bioactive food components, with nutrigenomic effects, contrast oxidative stress, mitochondrial dysfunction, autophagy and increasing genomic instability (63). Thus, considering this concept, it is reasonable to hypothesize that nutrients-regulated phenomena, at genomic and metabolic levels, can be the principal mechanisms by which it is possible to act for a homeostasis. All the dietary molecules able to change DNA methylation by exerting an epigenetic effect can be the actors of this mechanism of action. Methyl donor S-adenosylmethionine, methyltransferase inhibitor S-adenosylhomocysteine, and also the DNMTs (DNA Methyl Transferase)–TET (Ten-Eleven-Tlaslocation) enzymes can be targeted by diet molecules and the resulting change in DNA methylation can activate/silence genes implicated in some pathways, for example autophagy or inflammation (64). The mechanism of action of a supercritical CO_2_ extract from *Calendula officinalis*, commonly known as marigold, can be taken as an example. It induces the expression of *BMP8B* (Bone morphogenetic protein 8 b) gene, which directs to an energetic disaster ending up with autophagy-induced cell death (65).

Although nutrient availability strongly impacts the process of autophagy, the specific metabolites that regulate autophagy responses have not yet been determined. (S-adenosyl-l-methionine) SAM is the sole methyl group donor involved in the methylation of DNA, RNA and histones, in the methionine starvation-induced autophagy by epigenetic effects. Moreover, the metabolites of SAM, such as homocysteine, glutathione, decarboxylated SAM and spermidine, also exert important influences on the regulation of autophagy. These new purposes add an important perspective regarding the basal mechanism and regulation of autophagy (66).

## 4 Autophagy and nutrigenomics in specific pathologies

### 4.1 Role of autophagy and nutritional genomic mechanisms in neurodegenerative conditions

Recent research has increasingly highlighted the significance of autophagy in neurodegenerative disease, such as Alzheimer’s, Parkinson’s, and Huntington’s (7). Proteins prone to forming aggregates in neurodegenerative diseases are among the materials degraded through the autophagy pathway.

Various factors can compromise autophagy, including genetic mutations, aging, environmental factors, and impaired lysosomal function. All these factors disrupt the autophagy machinery, leading to a buildup of protein aggregates and contributing to neuronal toxicity and cell death observed in neurodegenerative conditions (7). For instance, mutations in the WIPI4 (WDR45) (WD repeat domain 45) gene associated with autophagy lead to (β-propeller protein-associated neurodegeneration) BPAN, exemplifying a direct link between autophagy-related genes and disease manifestation. Various genetic variations linked to neurodegenerative illnesses impact multiple facets of the autophagy process. These variants influence the initiation control through interactions with Beclin 1, the encapsulation of materials within the autophagosome, their transport to lysosomes, and the degradative capacity of these lysosomes (7).

Nixon et al. (67) focused on Alzheimer’s disease (AD) and delve into the intricate relationship between amyloid precursor protein (APP) and endosomal-lysosomal dysfunction in the context of AD pathogenesis. They emphasizes that abnormalities in the endosomal-lysosomal system, where APP processing occurs, play a crucial role in the development and progression of AD. They showed that alterations in endosomes and lysosomes, characterized by impaired clearance mechanisms and dysfunctional lysosomal degradation, contribute to the accumulation of toxic amyloid-beta peptides, a hallmark feature of AD pathology.

In light of these features, addressing these cellular processes could be important for the development of effective therapeutic interventions. Some studies suggest that certain molecules found in the diet may play a role in slowing down or reducing the effects of neurodegenerative diseases.

Simonsen et al. (68) explored the correlation between elevated basal autophagy levels and improved resistance against neurodegeneration and aging. By manipulating autophagy through dietary means, it highlights the potential of enhancing autophagy processes to enhance longevity and resilience against oxidative stress in the nervous system, suggesting dietary interventions as a potential avenue for managing neurodegenerative conditions.

Recent scientific investigations have delved into understanding how certain dietary components influence autophagy processes, holding potential implications for therapeutic interventions and preventive strategies in neurodegenerative conditions. Studies such as Rubinsztein et al. (69) have explored the role of caloric restriction and its impact on autophagy, suggesting a potential link between dietary control and the modulation of autophagy pathways implicated in neurodegenerative diseases. Furthermore, investigations by Madeo et al. (6) into caloric restriction mimetics (CRM) and their effects on autophagy pathways offer insights into potential therapeutic avenues that mimic the benefits of dietary restrictions (7).

CRMs are compounds that mimic the effects of CR without requiring a decrease in food intake. The authors focused on different classes of CRMs, including mTOR inhibitors, sirtuin activators, and metformin, and their potential mechanisms of action in promoting health and longevity. Indeed, they also examine the role of CRMs in cellular processes such as autophagy, mitochondrial function, and cellular senescence, supporting the significant potential of pharmacological induction of autophagy as a viable and efficient strategy also against metabolic disorders.

These studies collectively underscore the significance of specific dietary components in influencing autophagy processes and hint at their potential as targeted interventions for managing or preventing neurodegenerative conditions.

Recently, studies investigated how specific dietary components, such as polyphenols and omega-3 fatty acids, impact autophagy processes in neurodegenerative conditions, potentially offering therapeutic.

Shirooie et al. (70) investigated the potential therapeutic role of omega-3 fatty acids in addressing neurodegenerative conditions through the modulation of mTOR pathways. mTOR is a central regulator that influences various cellular functions, including autophagy. When mTOR is activated, it inhibits autophagy, suppressing the cellular recycling process. Conversely, inhibiting mTOR or its signaling pathways typically leads to autophagy activation, promoting the clearance of damaged or dysfunctional cellular components. Some studies suggest that omega-3 may modulate mTOR activity, potentially inhibiting mTOR or altering its downstream signaling, which could indirectly impact autophagy processes. By modulating mTOR, omega-3 could potentially promote autophagy activity, facilitating the removal of toxic protein aggregates or damaged cellular components associated with neurodegenerative conditions. Shirooie et al. (70) examined the impact of omega-3 fatty acids on mTOR pathways, discussing preclinical and clinical evidence supporting their potential to modulate mTOR activity. It may propose omega-3 fatty acids as a promising avenue for therapeutic intervention because of their ability to influence mTOR signaling, potentially offering neuroprotective effects and mitigating neurodegenerative processes.

Chandrasekaran et al. (71) explored how polyphenols, commonly found in plant-based foods, may possess neuroprotective properties and investigated their potential impact on autophagy within neuronal cells. The modulation of autophagy-related pathways by polyphenols, suggest their ability to enhance the removal of harmful cellular components implicated in neurodegenerative diseases. Since the polyphenols’ mechanism of action in modulating autophagy involves pathways such as mTOR, AMPK, SIRT-1, and ERK. Polyphenols evidently boost autophagy protein levels, such as Beclin-1, microtubule-associated protein light chain (LC3-I and -II), and Sirtuin1 (SIRT1). This emphasizes the therapeutic potential of polyphenols as potential agents to mitigate neurodegeneration by influencing autophagy processes.

**FIGURE 2:**
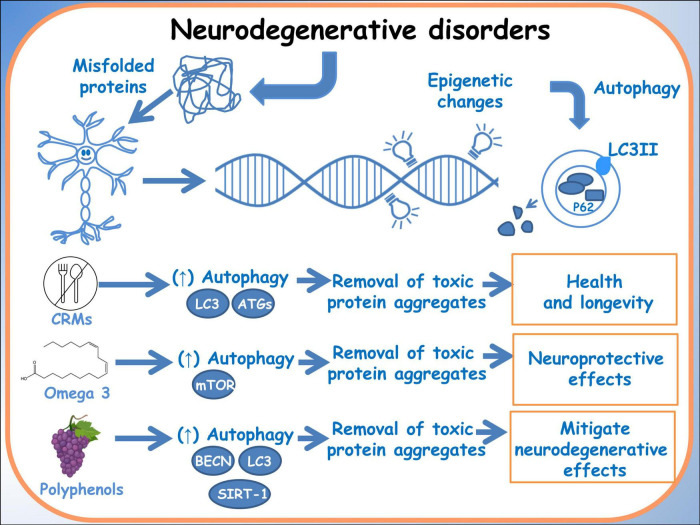
Autophagy in neurodegenerative disorders. A schematic diagram of the effects of dietary molecules on the genome and how they can influence autophagy, through epigenetic modifications.

The growing field of nutrigenomics holds promise in unveiling the intricate connections between dietary components and the molecular mechanisms behind neurodegenerative diseases. Specifically, the modulation of autophagy processes through dietary interventions emerges as a compelling avenue in mitigating neurodegeneration. Studies by Parrella et al. (72) and Wang et al. (73) have highlighted the relationship among dietary manipulations, autophagy and neurodegenerative diseases. Parrella et al. (72) elucidated that protein restriction cycles notably reduced phosphorylated Tau and IGF-1 levels while ameliorating behavioral performance in an Alzheimer’s mouse model, suggesting a potential modulation of autophagy. Moreover, Wang et al. (73) delved into the regulatory effects of omega-3 fatty acids and genetic variations on the cytoskeleton, offering insights into potential therapeutic avenues targeting autophagy pathways in Alzheimer’s disease. These findings underscore the significance of nutrigenomics interventions in modulating autophagy, providing a foundation for targeted dietary strategies aimed at combating neurodegeneration (Figure 2).

### 4.2 Implications of nutrigenomics in diabetes management and metabolic diseases via autophagic mechanisms

Autophagy, as a fundamental cellular process, is intricately linked to metabolic homeostasis and plays a critical role in diabetes pathogenesis, including insulin resistance and beta-cell dysfunction. The interplay between autophagy and the genomic effects of dietary compound holds promising implications for the management of diabetes and metabolic diseases.

Wang et al. (43) investigated the role of autophagy in regulating inflammation following oxidative injury in diabetes. They highlights the potential therapeutic implications of targeting autophagy pathways to mitigate inflammation and oxidative damage associated with diabetes (43). Autophagy plays a crucial role as a protective mechanism within cells. However, disturbances in autophagy are often linked to pathologies characterized by altered inflammatory responses. Specifically, Maedler et al. (74) demonstrated that defective autophagy-induced inflammation can lead to conditions such as obesity and insulin resistance. These defects in autophagy are believed to contribute to inflammation-associated metabolic diseases such as diabetes and obesity. Notably, studies indicate that cells undergoing autophagy-induced cell death can elicit a pro-inflammatory response, including the production and release of IL1B, which triggers a cascade of pro-inflammatory cytokines in human macrophages and β cells (74).

**FIGURE 3:**
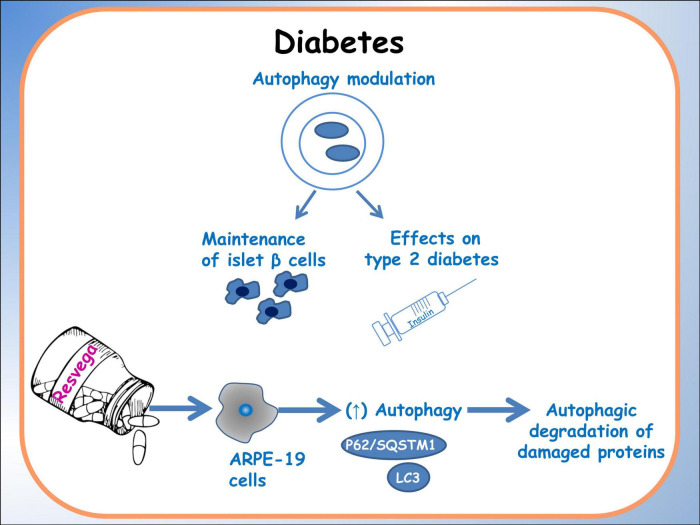
Autophagy in diabetes. A schematic diagram of the effects of dietary molecules on autophagy, particularly related to islet β cells. The main effects on the expression of autophagic factors are reported.

The integration of nutrigenomics, offers a personalized approach to metabolic disorders management. Noteworthy research, as discussed by Méndez et al. (75), indicates that specific dietary interventions, particularly those rich in polyphenols and omega-3 fatty acids, may enhance autophagy and alleviate metabolic stress. They explored the potential synergistic effects of polyphenols, supporting the combined use of these dietary components as nutraceuticals for managing metabolic disorders. They discuss the individual beneficial effects of polyphenols and fish oils, such as antioxidant and anti-inflammatory properties, as well as their potential to modulate lipid metabolism, glucose homeostasis, and insulin sensitivity. Additionally, the simultaneous consumption of polyphenols and fish oils may have enhanced metabolic benefits compared to their individual use. Indeed studies on Resvega, a commercial supplement containing resveratrol and omega-3 fatty acids, by Koskela and colleagues (76), demonstrated the ability to activate the autophagy machinery and increase autophagy in a starvation model. Additionally, Resvega contributed to the survival of ARPE-19 cells exposed to detrimental protein waste triggered by proteasome inhibition. The key regulator of autophagy, p62/SQSTM1 protein, showed increased levels under Resvega treatment, which contradicted the expected decrease during autophagy processes. This increase was attributed to Resvega’s omega-3 fatty acid content, which enhances the selectivity of damaged proteins for autophagy degradation. Resvega also significantly increased the level of LC3-II, indicating enhanced autophagosome formation, although the level decreased below baseline after prolonged treatment, suggesting active autophagy flux. The combination of Resvega with proteasome inhibitor MG-132 further enhanced autophagy flux. Furthermore, Resvega had a protective effect against harmful protein-induced cell death (Figure 3) (6).

### 4.3 Autophagy and genomic effects of dietary compound in cancer: therapeutic and preventive implications

Dysregulated autophagy has been implicated in various stages of cancer development, from initiation to metastasis, making it a promising target for cancer therapy (77, 78). Nutrigenomics explores, also, how dietary factors interact with the genome to modulate gene expression, influencing individual susceptibility to cancer and response to treatment (79). There is an extensive bibliography on how dietary components can regulate autophagy-related pathways, thus exerting both preventive and therapeutic effects against cancer (80–82). By targeting specific genes and signaling pathways involved in autophagy, these dietary compounds can either enhance the protective role of autophagy or induce cytotoxic autophagy cell death in cancer cells. Furthermore, dietary component may act as sensitizer for drug chemotherapy paving the way for further clinical exploration and application in cancer therapy.

Chen et al. (83) investigated the combination of epigallocatechin gallate (EGCG), a polyphenol found in green tea and Doxorubicin (DOX) in hepatocellular carcinoma (HCC) treatment. The study demonstrates that DOX induces autophagy in HCC cells, which may serve as a mechanism for cancer cell survival and evasion of chemotherapy-induced cytotoxicity. Inhibition of autophagy enhances the sensitivity of HCC cells to DOX, suggesting that targeting autophagy could improve chemotherapy outcomes. The authors demonstrated that EGCG suppresses basal autophagy activity in HCC cells in a dose-dependent manner, increasing cell death and apoptosis both *in vitro* and *in vivo*. The synergistic effect of EGCG and DOX is attributed to their complementary mechanisms of action. EGCG sensitizes tumor cells to DOX by downregulating multidrug resistance proteins and enhancing intracellular drug accumulation. Additionally, EGCG protects against DOX-induced cardiotoxicity, further highlighting its potential as a therapeutic agent in cancer treatment (83).

**FIGURE 4:**
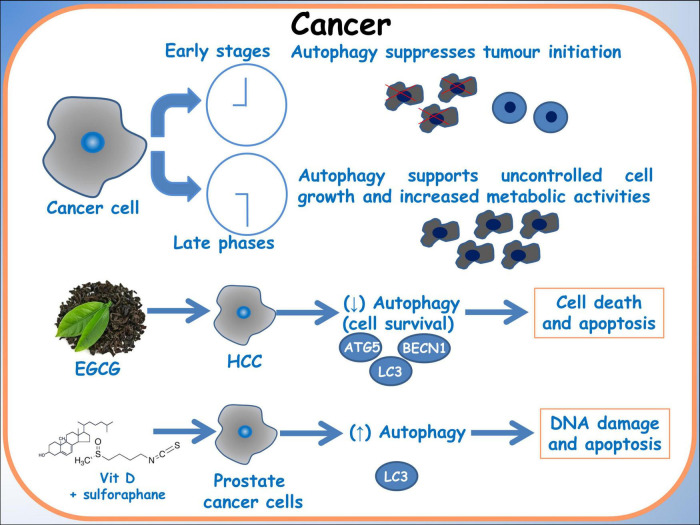
Autophagy in cancer. A schematic diagram of the effects of dietary molecules on cancer cells. In breast cancer cells, the effect on the autophagic gene BBCN1, related to BRCA1 expression is showed.

Furthermore, Tuttis et al. (57) discussed the potential of combining sulforaphane and vitamin D as a therapeutic strategy for advanced prostate cancer. These researches addressed challenges associated with conventional treatments, such as progression, metastasis, and drug resistance. The study conducted on prostate cancer cell lines DU145 and PC-3 reveals that while sulforaphane and vitamin D individually did not significantly affect cell viability, their combination led to decreased viability, modulation of apoptosis pathways, induction of autophagy and DNA damage. These effects were mediated by activation of the JNK/MAPK pathway. This study emphasizes the significance of comprehending pharmacodynamic interactions and recognizing the potential synergistic effects of dietary compounds as a personalized approach to cancer treatment (Figure 4) (61).

Taken together, the reported data highlight the pivotal role of autophagy and its modulation by natural compounds as a factor of remarkable importance in the characterization of specific diseases.

## 5 Conclusion

In conclusion, the intersection of genomic effect of dietary compound, autophagy, and disease pathology offers promising avenues for personalized therapeutic interventions and preventive strategies across various health conditions. Research into the role of autophagy in neurodegenerative diseases underscores the importance of understanding how dietary compounds can modulate this cellular process to mitigate disease progression. Studies exploring the impact of dietary interventions on autophagy pathways, such as omega-3 fatty acids and polyphenols, highlight their potential as neuroprotective agents. Additionally, the field of nutrigenomics provides insights into the intricate interactions between dietary components and the genome, offering opportunities for tailored dietary strategies to combat disease.

In cancer research, the dual role of autophagy presents challenges and opportunities for therapeutic interventions. Understanding how dietary compounds influence autophagy-related pathways in cancer cells offers avenues for both prevention and treatment. Combining phytochemicals such as sulforaphane and vitamin D demonstrates promising synergistic effects in targeting cancer cells. Investigating how dietary compounds can influence autophagy through epigenetic mechanisms provides promising directions for personalized cancer therapies, highlighting the significance of dietary interventions in treating cancer.

Overall, recognizing the complex interplay between autophagy, dietary interventions, and disease pathology underscores the importance of personalized approaches to healthcare. Nutrigenomics research holds significant potential in uncovering the mechanisms underlying these interactions, paving the way for targeted therapies and preventive measures tailored to individual patient needs.
